# Galectin-1 as a potential cancer target

**DOI:** 10.1038/sj.bjc.6602493

**Published:** 2005-03-22

**Authors:** G A Rabinovich

**Affiliations:** 1 Division of Immunogenetics, Hospital de Clínicas ‘José de San Martín’, University of Buenos Aires, Argentina

**Keywords:** galectin-1, apoptosis, tumour-immune escape

## Abstract

Galectins are a family of structurally related carbohydrate-binding proteins, which are defined by their affinity for poly-*N*-acetyllactosamine-enriched glycoconjugates and sequence similarities in the carbohydrate recognition domain. Galectin-1, a member of this family, contributes to different events associated with cancer biology, including tumour transformation, cell cycle regulation, apoptosis, cell adhesion, migration and inflammation. In addition, recent evidence indicates that galectin-1 contributes to tumour evasion of immune responses. Given the increased interest of tumour biologists and clinical oncologists in this field and the potential use of galectins as novel targets for anticancer drugs, we summarise here recent advances about the role of galectin-1 in different events of tumour growth and metastasis.

## 

### Classification and carbohydrate specificity

Galectins are animal lectins defined by shared consensus amino-acid sequences and affinity for *β*-galactose-containing oligosaccharides (Leffler *et al*, 2004). Members of the galectin family are composed of one or two carbohydrate-recognition domains (CRDs) of approximately 130 amino acids. Regarding the biochemical structure, some galectins contain one CRD and exist as monomers (galectin-5, -7 and -10) or dimers (galectin-1 ,-2, -11, -13, -14 and -15), whereas other galectins such as galectin-4, -6, -8, -9 and -12 contain two CRDs connected by a short linker region. In contrast, galectin-3 uniquely occurs as a chimeric protein with one CRD and an additional nonlectin domain, which is involved in the oligomerisation of this protein. It has been suggested that multivalency of individual members of the galectin family and their crosslinking properties might determine different biological responses by inducing aggregation of specific cell-surface glycoreceptors, which – in many cases – are associated with different signal transduction events (reviewed in Rabinovich *et al*, 2002a).

The first discovered protein in the family was galectin-1, a noncovalent dimer composed of subunits with one CRD. Although this protein binds preferentially to glycoconjugates containing the ubiquitous disaccharide *N*-acetyllactosamine (Gal *β*1-3/4 GlcNAc), binding to individual lactosamine units is of relatively low affinity and it is the arrangement of lactosamine disaccharides in repeating chains (polylactosamine) that increases the binding avidity (Schwarz *et al*, 1998; Ahmad *et al*, 2004).

### Subcellular distribution

Galectin-1 lacks recognisable secretion signal sequences and does not pass through the standard ER/Golgi pathway (Leffler *et al*, 2004). In addition, it shows characteristics of typical cytoplasmic proteins, including acetylated *N*-terminus and lack of glycosylation. However, there is evidence that this protein, as well as other members of the galectin family, is secreted by a novel mechanism distinct from classical vesicle-mediated exocytosis.

### Regulated expression of galectin-1 in tumours

Detailed description of the expression and functional status of galectins in different tumour types has been recently provided (Danguy *et al*, 2002; Nangia-Makker *et al*, 2002; van den Brüle *et al*, 2004; Lahm *et al*, 2004; Liu and Rabinovich, 2005). Here we will review the role of galectin-1 in different steps of tumour progression to evaluate its potential use as a therapeutic target in cancer.

Expression of galectin-1 has been well documented in many different tumour types including astrocytoma, melanoma and prostate, thyroid, colon, bladder and ovary carcinomas (reviewed by Danguy *et al*, 2002). Interestingly, in most cases such expression correlates with the aggressiveness of these tumours and the acquisition of metastatic phenotype. Whether expression of galectin-1 in tumour tissue or tumor-associated stroma may actively influence disease outcome still remains to be elucidated.

## GALECTIN-1 AND TUMOUR TRANSFORMATION

It has been recently demonstrated that intracellular galectin-1 may play a key role in the initiation of transformed phenotype of tumours. Kloog and colleagues have found that galectin-1 interacts with oncogenic H-RAS and contribute to membrane anchorage of H-RAS (Paz *et al*, 2001). Interestingly, overexpression of galectin-1 in tumour cells results in an increase in both the membrane association of H-RAS and cell transformation.

## GALECTIN-1 IN TUMOUR GROWTH

Over the past few years, the perceived role of galectin-1 in tumour growth has mirrored the story of Dr Jekyll and Mr Hide. While the endogenous protein may function as a growth-promoting factor, exogenously added galectin-1 specifically suppresses tumour cell proliferation. In this sense, Yamaoka *et al* (2000) showed that inhibition of *gal-1* gene expression in a rat glioma cell line arrests tumour growth, suggesting that endogenous galectin-1 has growth-promoting activity. On the other hand, Kopitz *et al* (2001) showed that exogenously added galectin-1 inhibits the growth of neuroblastoma cells. Thus, the effects of galectin-1 appear to be multifaceted. It can function in both carbohydrate-dependent and independent manners and its effects can be either positive or negative, depending on the responder cell types or its subcellular localisation. Interestingly, it has been reported that galectin-1 exerts a biphasic modulation of cell growth. While high doses of galectin-1 inhibit cell proliferation independent of its sugar-binding activity, low doses of galectin-1 are mitogenic and are susceptible to inhibition by lactose (Adams *et al*, 1996). Furthermore, galectin-1 can also regulate cell cycle progression in human tumour cells (Wells *et al*, 1999).

## GALECTIN-1 AND THE TUMOUR MICROENVIRONMENT

**Figure 1:**
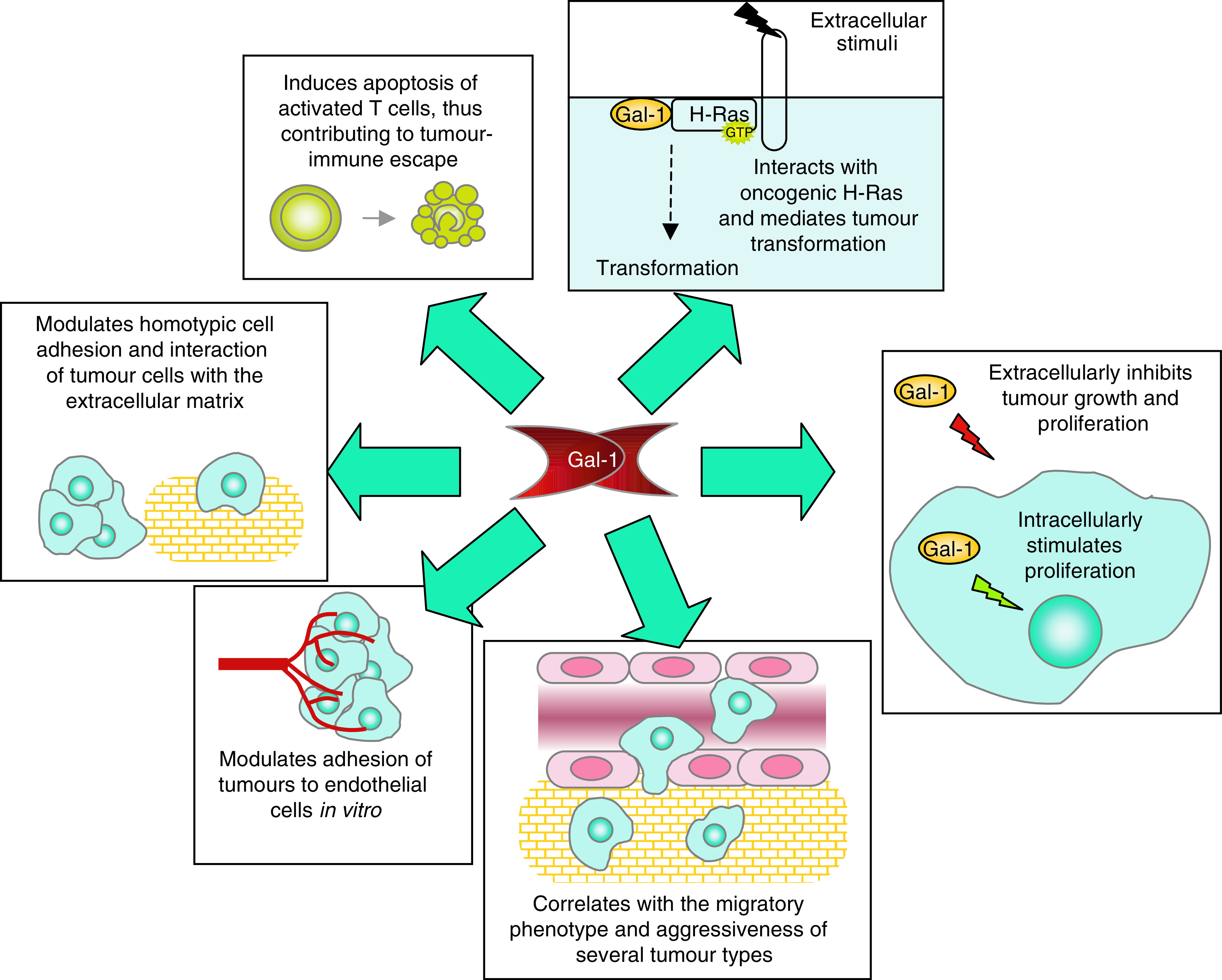
Contribution of galectin-1 to tumour progression. Galectin-1 interacts with oncogenic H-RAS and contributes to membrane anchorage of H-RAS and tumour transformation. In addition, this protein modulates cell growth, cell adhesion and cell migration, thereby affecting the process of tumour metastasis. Furthermore, recent evidence indicates that tumour cells secrete substantial levels of galectin-1 to evade T-cell-mediated responses.

Tumour metastasis is a multistep process that includes changes in cell adhesion, increased invasiveness, angiogenesis and evasion of the immune response. Galectin-1 has been shown to contribute to all these processes (Figure 1).

### Galectin-1 and cell adhesion

The metastatic cascade involves many changes in cell–cell and cell–extracellular matrix (ECM) interactions, and these include the detachment of cells from the primary tumour and their attachment to ECM proteins at distal sites. As they can bind to extracellular glycoconjugates, galectins might modulate the adhesion between adjacent cancer cells or between cancer cells and ECM. It has been shown that galectin-1 increases the adhesion of prostate and ovarian cancer cell lines to the ECM (Ellerhorst *et al*, 1999; van den Brüle *et al*, 2003). In addition, galectin-1 can also mediate homotypic cell aggregation of human melanoma cells in a carbohydrate-dependent manner (Tinari *et al*, 2001).

### Galectin-1 and the control of cell migration

Galectin-1 has been shown to affect cell migration of tumours and influence their invasiveness. In fact, exogenously added galectin-1 causes increased motility of glioblastoma cells *in vitro* (Rorive *et al*, 2001; Camby *et al*, 2002). Although the precise mechanisms have not yet been elucidated, it is possible that galectin-1 may engage cell surface glycocoproteins involved in cell motility. In addition, Clausse *et al* (1999) showed that this protein is upregulated in capillaries associated with carcinoma cells and can mediate interactions between tumours and endothelial cells *in vitro*, suggesting a potential role for galectin-1 in modulating angiogenesis.

## GALECTIN-1, INFLAMMATION AND ANTITUMOUR RESPONSES

Chronic inflammation is considered to be one of the most important factors contributing to tumour progression. Although the immune system can reduce tumour incidence through immune-surveillance mechanisms (Dunn *et al*, 2004), it can also promote tumour progression through inflammation-dependent mechanisms (Lin and Pollard, 2004). Galectins are expressed by many different inflammatory cells and regulate the function of these cells (Rabinovich *et al*, 2002a). In addition, galectins are released by tumours and can positively or negatively influence a variety of inflammatory responses.

### Galectin-1 and the inflammatory response

Undoubtedly, the most studied function for galectin-1 is related to the regulation of the inflammatory response. In recent years, it has become increasingly clear that galectin-1 can function as a homeostatic agent by modulating innate and adaptive immune responses. Galectin-1 induces cell growth inhibition, inhibits T-cell activation and promotes apoptosis of activated T cells (Perillo *et al*, 1995; Blaser *et al*, 1998; Rabinovich *et al*, 1998; Chung *et al*, 2000). Furthermore, we have recently shown that galectin-1 sensitises resting T cells to CD95/Fas-mediated cell death (Matarrese *et al*, 2005).

One concern regarding the proapoptotic activity of galectin-1 is that this effect has been demonstrated in most cases at relatively high concentrations (micromolar range) and it is uncertain whether high levels of soluble protein can be achieved *in vivo*. Interestingly, recent evidence indicates that the amount of galectin-1 secreted by different cell types is sufficient to kill T cells, when galectin-1 is presented in the context of the ECM (He and Baum, 2004).

Different cell surface glycoconjugates on the surface of activated T cells appear to be primary receptors for galectin-1, including CD45, CD43 and CD7 (Pace *et al*, 1999). Interestingly, galectin-1 binding to T cells results in marked redistribution of many of these glycoreceptors into segregated membrane microdomains. It has been demonstrated that the regulated expression of glycosyltransferases during development and activation, creating *N*-acetyllactosamine ligands, may determine T-cell susceptibility to galectin-1-induced cell death (Galvan *et al*, 2000; Amano *et al*, 2003).

As previously mentioned, CD7 has been identified as a critical receptor for galectin-1-induced apoptosis, and it has been recently demonstrated, that CD7^−^ T cells from patients with mycosis fungoides/Sezari syndrome are protected from galectin-1-mediated apoptosis (Rappl *et al*, 2002; Roberts *et al*, 2003).

The signal transduction events leading to galectin-1-induced apoptosis involve several intracellular mediators of apoptosis in primary T lymphocytes, including the induction of specific transcription factors, activation of caspases, cytochrome *c* release and participation of the ceramide pathway (Rabinovich *et al*, 2000a; Matarrese *et al*, 2005). However, a recent study showed that apoptosis induced by galectin-1 in a T-cell line is not dependent on the activation of caspase-3 or on cytochrome *c* release (Hahn *et al*, 2004). Furthermore, Dias-Baruffi *et al* (2003) reported that galectin-1 can induce the exposure of phosphatidylserine (an early apoptotic marker involved in the phagocytosis of apoptotic cells) on the plasma membrane of human T leukaemia cells and neutrophils, but this event does not result in DNA fragmentation. Thus, galectin-1 might activate different death pathways or different apoptosis end points in different cell types.

The pathophysiological relevance of galectin-1-induced cell death has been demonstrated in experimental models of chronic inflammation, including collagen-induced arthritis (Rabinovich *et al*, 1999b), inflammatory bowel disease (Santucci *et al*, 2003) and graft-versus-host disease (Baum *et al*, 2003). Interestingly, administration of galectin-1 *in vivo* suppresses Th1-dependent responses in these murine models and increases T-cell susceptibility to activation-induced cell death.

While relatively high concentrations of galectin-1 are required to promote T-cell apoptosis, we have demonstrated that galectin-1 at low concentrations (nanomolar range) provides a stop signal for T-cell adhesion to ECM and abrogates the production of proinflammatory cytokines, such as tumour necrosis factor-*α* (TNF-*α*) and interferon-*γ* (IFN-*γ*) by activated T cells, with no evidence of T-cell apoptosis (Rabinovich *et al*, 1999a). This observation supports the concept that this protein might also exert its anti-inflammatory effects through alternative nonapoptotic mechanisms. In addition, galectin-1 can also modulate acute inflammatory processes (Rabinovich *et al*, 2000b; Almkvist *et al*, 2002).

### Galectin-1 and tumour-immune escape

Despite the existence of specific T lymphocytes recognising tumour cells, the impact of these cells in tumour growth has been so far elusive. In contrast, several mechanisms have been described that potentially contribute to tumour cell evasion of the immune response (Dunn *et al*, 2004). These include the production of immunosuppressive cytokines and other soluble factors, including transforming growth factor-*β* (TGF-*β*), interleukin 10 (IL-10) and vascular endothelial growth factor (VEGF).

The immunoregulatory effects of galectin-1 and the correlation between galectin-1 expression in cancer cells and the aggressiveness of these tumours prompted us to investigate the role of galectin-1 in tumor-immune escape. We hypothesised that tumour cells may impair T-cell effector functions through secretion of galectin-1 and that this mechanism may contribute in tilting the balance towards an immunosuppressive environment at the tumour site. By a combination of *in vitro* and *in vivo* experiments using knockdown transfectants, we established a link between galectin-1-mediated immunoregulation and its contribution to tumour-immune escape (Rubinstein *et al*, 2004). Blockade of the inhibitory effects of galectin-1 within tumour tissue resulted in reduced tumour mass (an effect which required intact CD4^+^ and CD8^+^ T-cell responses) and stimulated the generation of a tumour-specific T-cell response *in vivo*. Our observations support the idea that galectin-1 may contribute to immune privilege of tumours by modulating survival or polarisation of effector T cells, and suggest a potential molecular target for manipulation of T-cell apoptosis with potential implications in immunotherapy.

## GALECTIN-1 AS A TARGET FOR ANTICANCER AGENTS: CONCLUSIONS AND PERSPECTIVES

Given the contribution of galectin-1 to tumour growth and metastasis, it is predicted that inhibitors of galectin-1 will find their way into cancer clinical trials, leading to delays in tumour progression and improvements in overall survival. Challenges for the future will be to employ potent and selective small inhibitors of galectin-1 and, in fact, molecules with such properties have already been developed for galectin-1 or other galectins (Andre *et al*, 2001; Sorme *et al*, 2002). Furthermore, galectin-1 expression can be modulated by chemotherapeutic and antimetastatic agents (Lu *et al*, 2000; Rabinovich *et al*, 2002b). A current challenge is the design of more specific and potent galectin-1 inhibitors for therapeutic purposes with no or minimal adverse effects. Although galectin-1 still remains elusive in terms of our understanding of its multifunctional modes of action, we are moving ever closer to unravelling this mystery at a molecular level and to design new therapeutic approaches directed toward modulating its activities.

## References

[bib1] Adams L, Scott GK, Weinberg CS (1996) Biphasic modulation of cell growth by recombinant human galectin-1. Biochim Biophys Acta 1312: 137–144 8672536 10.1016/0167-4889(96)00031-6

[bib2] Ahmad N, Gabius HJ, Sabesan S, Oscarson S, Brewer CF (2004) Thermodynamic binding studies of bivalent oligosaccharides to galectin-1, galectin-3 and the carbohydrate recognition domain of galectin-3. Glycobiology 14: 817–825 15148296 10.1093/glycob/cwh095

[bib3] Almkvist J, Dahlgren C, Leffler H, Karlsson A (2002) Activation of the neutrophil nicotinamide adenine dinucleotide phosphatase oxidase by galectin-1. J Immunol 168: 4034–4041 11937561 10.4049/jimmunol.168.8.4034

[bib4] Amano M, Galvan M, He J, Baum LG (2003) The ST6Gal I sialyltransferase selectively modifies *N*-glycans on CD45 to negatively regulate galectin-1-induced CD45 clustering, phosphatase modulation, and T cell death. J Biol Chem 278: 7469–7475 12499376 10.1074/jbc.M209595200

[bib5] Andre S, Pieters RJ, Vrasidas I, Kaltner H, Kuwabara I, Liu FT, Liskamp RM, Gabius HJ (2001) Wedgelike glycodendrimers as inhibitors of binding of mammalian galectins to glycoproteins, lactose maxiclusters, and cell surface glycoconjugates. Chembiochem 2: 822–830 11948868 10.1002/1439-7633(20011105)2:11<822::AID-CBIC822>3.0.CO;2-W

[bib6] Baum LG, Blackall DP, Arias-Magallano S, Nanigian D, Uh SY, Browne JM, Hoffmann D, Emmanouilides CE, Territo MC, Baldwin GC (2003) Amelioration of graft *versus* host disease by galectin-1. Clin Immunol 109: 295–307 14697744 10.1016/j.clim.2003.08.003

[bib7] Blaser C, Kaufmann M, Muller C, Zimmermann C, Wells V, Mallucci L, Pircher H (1998) Beta-galactoside-binding protein secreted by activated T cells inhibits antigen-induced proliferation of T cells. Eur J Immunol 28: 2311–2319 9710209 10.1002/(SICI)1521-4141(199808)28:08<2311::AID-IMMU2311>3.0.CO;2-G

[bib8] Camby I, Belot N, Lefranc F, Sadeghi N, de Launoit Y, Kaltner H, Musette S, Darro F, Danguy A, Salmon I, Gabius HJ, Kiss R (2002) Galectin-1 modulates human glioblastoma cell migration into the brain through modifications to the actin cytoskeleton and levels of expression of small GTPases. J Neuropathol Exp Neurol 61: 585–596 12125737 10.1093/jnen/61.7.585

[bib9] Chung CD, Patel VP, Moran M, Lewis LA, Miceli MC (2000) Galectin-1 induces partial TCR beta-chain phosphorylation and antagonizes processive TCR signal transduction. J Immunol 165: 3722–3729 11034377 10.4049/jimmunol.165.7.3722

[bib10] Clausse N, van den Brule F, Waltregny D, Garnier F, Castronovo V (1999) Galectin-1 expression in prostate tumor-associated capillary endothelial cells is increased by prostate carcinoma cells and modulates heterotypic cell–cell adhesion. Angiogenesis 3: 317–325 14517411 10.1023/a:1026584523789

[bib11] Danguy A, Camby I, Kiss R (2002) Galectins and cancer. Biochim Biophys Acta 1572: 285–293 12223276 10.1016/s0304-4165(02)00315-x

[bib12] Dias-Baruffi M, Zhu H, Cho M, Karmakar S, McEver RP, Cummings RD (2003) Dimeric galectin-1 induces surface exposure of phosphatidylserine and phagocytic recognition of leukocytes without inducing apoptosis. J Biol Chem 278: 41282–41293 12853445 10.1074/jbc.M306624200

[bib13] Dunn GP, Old LJ, Schreiber RD (2004) The immunobiology of cancer immunosurveillance and immunoediting. Immunity 21: 137–148 15308095 10.1016/j.immuni.2004.07.017

[bib14] Ellerhorst J, Nguyen T, Cooper DN, Lotan D, Lotan R (1999) Differential expression of endogenous galectin-1 and galectin-3 in human prostate cancer cell lines and effects of overexpressing galectin-1 on cell phenotype. Int J Oncol 14: 217–224 9917495

[bib15] Galvan M, Tsuboi S, Fukuda M, Baum LG (2000) Expression of a specific glycosyltransferase enzyme regulates T cell death mediated by galectin-1. J Biol Chem 275: 16730–16737 10747988 10.1074/jbc.M001117200

[bib16] Hahn HP, Pang M, He J, Hernandez JD, Yang RY, Li LY, Wang X, Liu FT, Baum LG (2004) Galectin-1 induces nuclear translocation of endonuclease G in caspase- and cytochrome *c*-independent T cell death. Cell Death Differ 11: 1277–1286 15297883 10.1038/sj.cdd.4401485 PMC1201488

[bib17] He J, Baum LG (2004) Presentation of galectin-1 by extracellular matrix triggers T cell death. J Biol Chem 279: 4705–4712 14617626 10.1074/jbc.M311183200

[bib18] Kopitz J, von Reitzenstein C, Andre S, Kaltner H, Uhl J, Ehemann V, Cantz M, Gabius HJ (2001) Negative regulation of neuroblastoma cell growth by carbohydrate-dependent surface binding of galectin-1 and functional divergence from galectin-3. J Biol Chem 276: 35917–35923 11451961 10.1074/jbc.M105135200

[bib19] Lahm H, Andre S, Hoeflich A, Kaltner H, Siebert HC, Sordat B, von der Lieth CW, Wolf E, Gabius HJ (2004) Tumor galectinology: insights into the complex network of a family of endogenous lectins. Glycoconj J 20: 227–238 15115907 10.1023/B:GLYC.0000025817.24297.17

[bib20] Leffler H, Carlsson S, Hedlund M, Qian Y, Poirier F (2004) Introduction to galectins. Glycoconj J 19: 433–440 10.1023/B:GLYC.0000014072.34840.04 14758066

[bib21] Lin EY, Pollard JW (2004) Role of infiltrated leucocytes in tumour growth and spread. Br J Cancer 90: 2053–2058 15164120 10.1038/sj.bjc.6601705 PMC2410285

[bib22] Liu F, Rabinovich G (2005) Galectins as modulators of tumour progression. Nat Rev Cancer 5: 29–41 15630413 10.1038/nrc1527

[bib23] Lu Y, Lotan D, Lotan R (2000) Differential regulation of constitutive and retinoic acid-induced galectin-1 gene transcription in murine embryonal carcinoma and myoblastic cells. Biochim Biophys Acta 1491: 13–19 10760565 10.1016/s0167-4781(00)00055-5

[bib24] Matarrese P, Tinari A, Mormone E, Bianco GA, Toscano MA, Ascione B, Rabinovich GA, Malorni W (2005) Galectin-1 sensitizes resting human T lymphocytes to Fas (CD95)-mediated cell death via mitochondrial hyperpolarization, budding and fission. J Biol Chem 280: 6969–6985 15556941 10.1074/jbc.M409752200

[bib25] Nangia-Makker P, Conklin J, Hogan V, Raz A (2002) Carbohydrate-binding proteins in cancer, and their ligands as therapeutic agents. Trends Mol Med 8: 187–192 11927277 10.1016/s1471-4914(02)02295-5

[bib26] Pace KE, Lee C, Stewart PL, Baum LG (1999) Restricted receptor segregation into membrane microdomains occurs on human T cells during apoptosis induced by galectin-1. J Immunol 163: 3801–3811 10490978

[bib27] Paz A, Haklai R, Elad-Sfadia G, Ballan E, Kloog Y (2001) Galectin-1 binds oncogenic H-Ras to mediate Ras membrane anchorage and cell transformation. Oncogene 20: 7486–7493 11709720 10.1038/sj.onc.1204950

[bib28] Perillo NL, Pace KE, Seilhamer JJ, Baum LG (1995) Apoptosis of T cells mediated by galectin-1. Nature 378: 736–739 7501023 10.1038/378736a0

[bib29] Rabinovich GA, Alonso CR, Sotomayor CE, Durand S, Bocco JL, Riera CM (2000a) Molecular mechanisms implicated in galectin-1-induced apoptosis: activation of the AP-1 transcription factor and downregulation of Bcl-2. Cell Death Differ 7: 747–753 10918449 10.1038/sj.cdd.4400708

[bib30] Rabinovich GA, Ariel A, Hershkoviz R, Hirabayashi J, Kasai KI, Lider O (1999a) Specific inhibition of T-cell adhesion to extracellular matrix and proinflammatory cytokine secretion by human recombinant galectin-1. Immunology 97: 100–106 10447720 10.1046/j.1365-2567.1999.00746.x PMC2326819

[bib31] Rabinovich GA, Baum LG, Tinari N, Paganelli R, Natoli C, Liu FT, Iacobelli S (2002a) Galectins and their ligands: amplifiers, silencers or tuners of the inflammatory response? Trends Immunol 23: 313–320 12072371 10.1016/s1471-4906(02)02232-9

[bib32] Rabinovich GA, Daly G, Dreja H, Tailor H, Riera CM, Hirabayashi J, Chernajovsky Y (1999b) Recombinant galectin-1 and its genetic delivery suppress collagen-induced arthritis via T cell apoptosis. J Exp Med 190: 385–398 10430627 10.1084/jem.190.3.385 PMC2195592

[bib33] Rabinovich GA, Iglesias MM, Modesti NM, Castagna LF, Wolfenstein-Todel C, Riera CM, Sotomayor CE (1998) Activated rat macrophages produce a galectin-1-like protein that induces apoptosis of T cells: biochemical and functional characterization. J Immunol 160: 4831–4840 9590230

[bib34] Rabinovich GA, Rubinstein N, Matar P, Rozados V, Gervasoni S, Scharovsky GO (2002b) The antimetastatic effect of a single low dose of cyclophosphamide involves modulation of galectin-1 and Bcl-2 expression. Cancer Immunol Immunother 50: 597–603 11807623 10.1007/s00262-001-0238-2 PMC11032852

[bib35] Rabinovich GA, Sotomayor CE, Riera CM, Bianco I, Correa SG (2000b) Evidence of a role for galectin-1 in acute inflammation. Eur J Immunol 30: 1331–1339 10820379 10.1002/(SICI)1521-4141(200005)30:5<1331::AID-IMMU1331>3.0.CO;2-H

[bib36] Rappl G, Abken H, Muche JM, Sterry W, Tilgen W, Andre S, Kaltner H, Ugurel S, Gabius HJ, Reinhold U (2002) CD4+CD7− leukemic T cells from patients with Sezary syndrome are protected from galectin-1-triggered T cell death. Leukemia 16: 840–845 11986945 10.1038/sj.leu.2402438

[bib37] Roberts AA, Amano M, Felten C, Galvan M, Sulur G, Pinter-Brown L, Dobbeling U, Burg G, Said J, Baum LG (2003) Galectin-1-mediated apoptosis in mycosis fungoides: the roles of CD7 and cell surface glycosylation. Mod Pathol 16: 543–551 12808059 10.1097/01.MP.0000071840.84469.06

[bib38] Rorive S, Belot N, Decaestecker C, Lefranc F, Gordower L, Micik S, Maurage CA, Kaltner H, Ruchoux MM, Danguy A, Gabius HJ, Salmon I, Kiss R, Camby I (2001) Galectin-1 is highly expressed in human gliomas with relevance for modulation of invasion of tumor astrocytes into the brain parenchyma. Glia 33: 241–255 11241742 10.1002/1098-1136(200103)33:3<241::aid-glia1023>3.0.co;2-1

[bib39] Rubinstein N, Alvarez M, Zwirner NW, Toscano MA, Ilarregui JM, Bravo A, Mordoh J, Fainboim L, Podhajcer OL, Rabinovich GA (2004) Targeted inhibition of galectin-1 gene expression in tumor cells results in heightened T cell-mediated rejection; a potential mechanism of tumor-immune privilege. Cancer Cell 5: 241–251 15050916 10.1016/s1535-6108(04)00024-8

[bib40] Santucci L, Fiorucci S, Rubinstein N, Mencarelli A, Palazzetti B, Federici B, Rabinovich GA, Morelli A (2003) Galectin-1 suppresses experimental colitis in mice. Gastroenterology 124: 1381–1394 12730878 10.1016/s0016-5085(03)00267-1

[bib41] Schwarz FP, Ahmed H, Bianchet MA, Amzel LM, Vasta GR (1998) Thermodynamics of bovine spleen galectin-1 binding to disaccharides: correlation with structure and its effect on oligomerization at the denaturation temperature. Biochemistry 37: 5867–5877 9558320 10.1021/bi9716478

[bib42] Sorme P, Qian Y, Nyholm PG, Leffler H, Nilsson UJ (2002) Low micromolar inhibitors of galectin-3 based on 3′-derivatization of *N*-acetyllactosamine. Chembiochem 3: 183–189 11921396 10.1002/1439-7633(20020301)3:2/3<183::aid-cbic183>3.0.co;2-#

[bib43] Tinari N, Kuwabara I, Huflejt ME, Shen PF, Iacobelli S, Liu FT (2001) Glycoprotein 90K/MAC-2BP interacts with galectin-1 and mediates galectin-1-induced cell aggregation. Int J Cancer 91: 167–172 11146440 10.1002/1097-0215(200002)9999:9999<::aid-ijc1022>3.3.co;2-q

[bib44] van den Brüle F, Califice S, Castronovo V (2004) Expression of galectins in cancer: a critical review. Glycoconj J 19: 537–542 10.1023/B:GLYC.0000014083.48508.6a 14758077

[bib45] van den Brüle F, Califice S, Garnier F, Fernandez PL, Berchuck A, Castronovo V (2003) Galectin-1 accumulation in the ovary carcinoma peritumoral stroma is induced by ovary carcinoma cells and affects both cancer cell proliferation and adhesion to laminin-1 and fibronectin. Lab Invest 83: 377–386 12649338 10.1097/01.lab.0000059949.01480.40

[bib46] Wells V, Davies D, Mallucci L (1999) Cell cycle arrest and induction of apoptosis by beta galactoside binding protein (beta GBP) in human mammary cancer cells. A potential new approach to cancer control. Eur J Cancer 35: 978–983 10533482 10.1016/s0959-8049(99)00020-9

[bib47] Yamaoka K, Mishima K, Nagashima Y, Asai A, Sanai Y, Kirino T (2000) Expression of galectin-1 mRNA correlates with the malignant potential of human gliomas and expression of antisense galectin-1 inhibits the growth of 9 glioma cells. J Neurosci Res 59: 722–730 10700009 10.1002/(SICI)1097-4547(20000315)59:6<722::AID-JNR4>3.0.CO;2-H

